# Water-Soluble Total Flavonoids Isolated from *Isodon lophanthoides* var*. gerardianus* (Benth.) H. Hara Promote Hepatocellular Carcinoma Sensitivity to 5-Fluorouracil

**DOI:** 10.1155/2021/6623212

**Published:** 2021-07-17

**Authors:** Chuan-Ping Feng, Hai-Xia Ding, Ying-Xin Liu, Qing-Feng Di, Yan Liu, Jian Liang, Guang-Xi Liu

**Affiliations:** ^1^Hunan Traditional Chinese Medical College, Zhuzhou 412012, Hunan, China; ^2^The First Affiliated Hospital of Hunan Traditional Chinese Medical College, Zhuzhou 412012, Hunan, China; ^3^School of Pharmaceutical Sciences, Guangzhou University of Chinese Medicine, Guangzhou 510006, China

## Abstract

*Isodon lophanthoides* var*. gerardianus* (Benth.) H. Hara, a native medicinal plant produced chiefly across Southern China, is one of the mainstream varieties of Xihuangcao, which has long been applied in preventing and treating some common liver or gall diseases. Water-soluble total flavonoids (WSTF) extracted from folk herbal medicine have many pharmacological effects. The objective of the paper is to investigate the synergy of WSTF with 5-ﬂuorouracil (5-FU) on HCC and the related mechanisms. Cells were exposed to WSTF alone or combination treatment with 5-FU. Then, in this study, we conducted cell viability test, cell cycle and clone forming test, apoptosis assay, reactive oxygen species (ROS), Western blotting, immunohistochemistry, and a xenograft tumor growth model for investigating the role of WSTF in HCC *in vivo* and *in vitro*. It was discovered that WSTF caused cell cycle arrest at the *G*0/*G*1 phase while increasing the ROS contents. The generation of ROS levels could cause cell apoptosis and inhibit colony formation. WSTF decreased the Bcl-2 level but promoted the Bax level. These showed the mitochondrial dependence of WSTF-mediated apoptosis. WSTF combined with 5-FU have a synergistic effect to significantly inhibit carcinogenicity *in vivo* and *in vitro*. The reduced ROS changed the synergy of WSTF with 5-FU. At last, WSTF inhibit the growth of HCC and promote the HCC sensitivity to 5-FU through ROS accumulation. WSTF-increased ROS levels may partially or completely contribute to enhanced toxicity. WSTF combined with 5-FU in HCC can play a synergistic effect when applied in the clinical setting.

## 1. Introduction

Hepatocellular carcinoma (HCC) has become a leading cause of cancer death worldwide (No. 2 in China). It tends to be diagnosed at advanced stages and has high morbidity and high mortality [1, 2], poor prognosis [3], and high rates of recurrence and metastasis [4]. For these reasons, liver cancer patients were forced to accept surgical resection to be the optimal treatment for HCC in China. More unfortunately, recurrence and metastasis created new difficulties for them following surgical treatment. As a result, a number of studies have been conducted to discover novel effective agents to reduce the recurrence and metastasis of the tumor and to improve symptoms together with patient survival and quality of life.

*Isodon lophanthoides* var. *gerardianus* (Benth.) H. Hara belongs to the Lamiaceae family [5], and as one of the mainstream varieties of Xihuangcao, this autochthonous plant is produced chiefly in South China. It is one of the traditional Chinese medicine (TCM) herbs extensively utilized to prevent and treat common gall or liver diseases, such as acute cholecystitis and acute jaundice hepatitis [6–8]. Clinical studies showed that prescription based on folk herbal medicine can improve the effective quality of life of HCC patients [9]. Clinical studies in the treatment of moderate or advanced liver cancer also showed that *Isodon lophanthoides* var. *gerardianus* can improve significantly patients' general symptoms to prolong their lives [10]. The autochthonous plant is characterized by low toxicity and broad pharmacological activities, including anti-inflammation, antioxidation, anticancer, and liver protection [11–19]. Studies showed that the water extracts from traditional Chinese medicine had significant inhibitory effects on HepG2 cell growth *in vitro* [20, 21] and the ethyl acetate extract from the plant had antitumor cell activity [22]. The water extract of the plant can inhibit tumor growth in mice bearing liver cancer H22 [23]. WSTF suppress HepG2 cell growth [24]. More recently, as suggested in our study, WSTF caused the apoptosis of HepG2 cells by increasing ROS, releasing mitochondrial cytochrome c into the cytoplasm, downregulating survivin and Bcl-2 levels, decreasing Bax and caspase-3 levels [25]. Based on the above results, WSTF are the candidate agent to treat HCC.

5-ﬂuorouracil (5-FU) has been extensively utilized to treat HCC [26], colorectal cancer (CRC), and gastric cancer (GC) [27]. 5-FU can arrest the cell cycle and the subsequent associated apoptosis, which is related to the inhibition of nucleoside metabolism. The accumulation of ROS in MCF-7 cells enhances the chemosensitivity of 5-FU [28]. WSTF elevate ROS levels in HepG2 cells [25], whether WSTF play a synergistic effect of 5-FU on HCC is not clear.

In this work, we detected how WSTF affected HCC alone or in combination with 5-FU both *in vivo* and *in vitro*. As a result, WSTF enhanced the HCC chemotherapeutic sensitivity to 5-fluorouracil in HCC *in vivo* and *in vitro*. WSTF plus 5-FU can play a synergistic effect on HCC when applied in the clinical setting.

## 2. Materials and Methods

### 2.1. Herbal Medicines and Extraction

**Figure 1:**
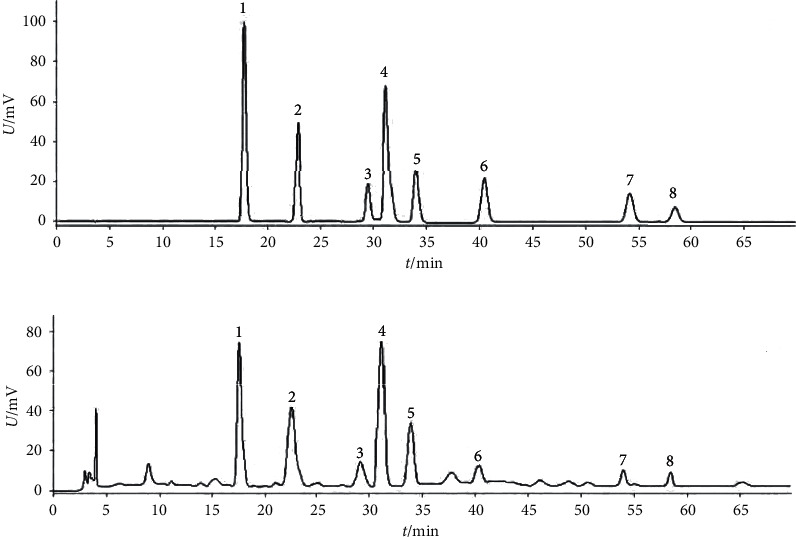
High-performance liquid chromatography of standard reference and WSFT samples. (a) A mixture of standard references and (b) WSTF—1: caffeic acid; 2: vicenin II; 3: vicenin III; 4: isoschaftoside; 5: schaftoside; 6: vitexin; 7: 6,8-di-C-a-L-arabinosylapigenin; 8: rutin.

*Isodon lophanthoides* var. *gerardianus* (Benth.) H. Hara (Batch No.: 20141022) was provided by Guangzhou Baiyun Mountain and Hutchison Whampoa Ltd. and confirmed by Professor Jian-nan Chen from the Guangzhou University of Chinese Medicine, China. After being appropriately chopped, the proper amounts of medicinal materials were extracted three times with distilled water and filtered. After vacuum concentration and resuspension within distilled water, the HPD-100 macroporous adsorptive resin column, as well as the polyamide column, was used to separate the components successively. Then, we used a vacuum to dry the 30% ethanol eluate into brown powder (namely, water-soluble total flavonoids, referred to as “WSTF”). Thereafter, high-performance liquid chromatography (HPLC) analysis was performed for WSTF (Figure 1). There were eight water-soluble components here, which are as follows: vicenin II (3.91 mg/g), caffeic acid (0.63 mg/g), schaftoside (3.01 mg/g), isoschaftoside (7.18 mg/g), vicenin I (0.45 mg/g), vitexin (0.53 mg/g), rutin (0.54 mg/g), and 6,8-di-C-a-L-arabinosylapigenin (0.57 mg/g) [25].

### 2.2. Cell Cultures

The HepG2 and SMMC-7721 cells were provided by the Shanghai Institute of Life Sciences, Chinese Academy of Sciences (Shanghai, China), and cultivated within the RPMI medium 1640 (Gibco, Grand Island, NY, USA) basic containing 10% (v/v) fetal bovine serum (FBS; Gibco, Melbourne, Australia) and 100 U/mL penicillin together with 100 *µ*g/mL streptomycin in a humid incubator under 5% CO_2_ conditions at 37°C. The medium was renewed two times/week.

### 2.3. Cytotoxicity Assays

HepG2 cells (5 × 10^3^ each well) along with SMMC-7721 cells (5 × 10^3^ each well) that underwent exponential growth were planted into 96-well plates followed by WSTF treatment at various doses for different times to plot the curve of dose as a function of time. MTT assay was used to assess cell viability. SPSS was employed to calculate IC_50_.

### 2.4. Cell Cycle Analysis

HepG2 cells (5 × 10^5^ cells/well) or SMMC-7721 cells (5 × 10^5^ cells/well) achieving logarithmic growth were planted into 6-well plates followed by 24 h of serum starving. After 48 h of WSTF treatment, cells were digested with trypsin and collected followed by 2 h of fixation with 70% ice-cold ethanol. Thereafter, the freshly prepared propidium iodide (PI, 50 mg/mL) dye that contained 100 mg/mL RNase A was used to incubate the cells for 30 min in accordance with specific protocols (BestBio Biotech Company, Shanghai, China). Then, a total of 20,000 cells from each sample were analyzed using the FACS canto™ II flow cytometer.

### 2.5. Intracellular ROS Level

We used the ROS detection kit to measure intracellular ROS levels. First, HepG2 cells (1 × 10^5^ each well) were planted into 6-well plates with 24 h of serum starving. Treated with WSTF with or without 5 mM NAC (N-acetylcysteine) or 5-FU, the HepG2 cell line was subjected to 48 h of incubation. 100 *µ*M of DCFH-DA was used to incubate HepG2 cells following specific protocols (Beyotime Biotechnology, Shanghai, China), and PBS was used to wash for thrice. Finally, the fluorescence intensity was determined by the microplate reader.

### 2.6. Apoptosis Assay

HepG2 cells (5 × 10^5^ each well) were inoculated into 6-well plates under 24 h of serum starving, and then WSTF or 5-FU was used to treat HepG2 cells for a period of 48 h, respectively. The HepG2 cell line was harvested and dyed using the apoptosis detection kit in accordance with specific protocols (Nanjing Jiancheng Bioengineering Institute, Nanjing, China), and then flow cytometry was applied to analyze 10,000 cells from each sample.

### 2.7. Colony Formation Assay

Cells at logarithmic growth phase were inoculated into 6-well plates at 500/well. HepG2 cells were exposed to WSTF at the indicated doses or 5 mM NAC for a period of 48 h. Then, the medium was renewed every two days. Stained with 1% crystal violet, the plates were photographed 12 days later. The Alpha Innotech Imaging System (Alphatron Asia Pte Ltd., Singapore) was used to count colonies from each sample.

### 2.8. Western Blot Analysis

The HepG2 cell line was harvested and rinsed using PBS. According to the manufacturer's instructions, the RIPA lysis buffer supplemented with the complete protease inhibitor (Roche, Switzerland) was used to lyse HepG2 cells. 25 *µ*g of proteins was incubated and then was isolated onto 12% SDS-PAGE followed by transfer onto the PVDF membranes. Thereafter, the 3% skimmed dry milk powder was used to block PVDF membranes followed by incubation with the primary antibody at 4°C for 12 h according to the specifications [29]. Later, secondary IgG antibody (goat anti-mouse or anti-rabbit IgG) was used to incubate the membranes for another 2 h. After washing, the ECL kit (Amersham Pharmacia Biotech) was used to react with membranes. In the study, the following antibodies (San Diego, CA, USA) were utilized: PARP, cleaved PARP, Bax, Bcl-2, cytochrome c, AIF (all dilutions, 1 : 800), together with beta-actin (1 : 2000).

### 2.9. Efficacy of Drug Combination

For evaluating the efficacy of drug combination, we calculated the values of combination index (CI) and the values of dose-reduction index (DRI) through using CalcuSyn (Biosoft, Ferguson, MO, USA). CI represents the degree of drug interaction, where CI < 1 suggests a synergistic effect, CI = 1 represents an additive effect, and CI > 1 represents antagonism. DRI represents a reduced dose of every drug when used in the synergistic combination relative to that used alone at the given effect level. Typically, DRI > 1 shows a reduction in dosage and toxicity, respectively, after combination treatment.

### 2.10. Mechanism Research for the Synergistic Effect of WSTF Plus 5-FU on HCC Cell Apoptosis

HepG2 cells were subjected to Annexin V/PI staining for evaluating the WSTF and/or 5-FU-induced apoptosis according to Section 2.6. Western blot analysis for the expression of apoptosis-related proteins was completed after the combination of WSTF and 5-FU. HepG2 cells were subjected to Annexin V/PI staining for evaluating apoptosis induced by WSTF plus 5-FU with or without NAC. After 48 h of incubation with WSTF and 5-FU with or without NAC, the ROS level in HepG2 cells was determined by DCFH-DA, and simultaneously, the expression of apoptosis-related proteins by Western blotting was completed. Intracellular ROS levels were measured after the combination of WSTF and 5-FU according to Section 2.5.

### 2.11. Xenograft Tumorigenicity Assay

Male and female athymic BALB/c-nu/nu mice (18–22 g; 4–6 weeks old) were provided by the Laboratory Animal Center of the Guangzhou University of Chinese Medicine (Guangzhou, China) and maintained under specific pathogen-free conditions. All *in vivo* studies were carried out following the National Institutes of Health guidelines (NIH publication 86-23, revised 1985). Each experimental protocol related to animal or animal care gained approval from the Ethics Committee of Laboratory Animal Services Center of the Guangzhou University of Chinese Medicine (SCXK2013-0034). Humane care was provided for each mouse, and their suffering was minimized. After a week, each animal was given a subcutaneous injection of 200 *µ*L cell solution, which contained 2 × 10^6^ HepG2 cells at the exponential phase, into the oxter. Later, each animal was monitored every day for mental state, bowel function, and diet consumption, while the subcutaneous tumor size (length and width) was determined at intervals of 3 days using Vernier calipers. Then, tumor volume was determined according to *V*_tumor_ = length × width^2^ × 0.5. On day 6 after inoculation, nude mice were allocated randomly to five groups when the tumor volume was the size of 100 mm^3^. There were a normal control group, model group (blank control group, BC), WSTF group, 5-FU group, and combination group, 6 nude mice per group. Intervention was administered in each group as follows: the WSTF group was administered WSTF by gavage 200 mg/kg/d once daily; the 5-FU group was administered 5-fluorouracil (5-FU, 20 mg/kg/d) *via* intraperitoneal injection, twice a week, while NS was administered saline *via* lavage at some other time; the combination group (WSTF + 5-FU group) was administered WSTF by lavage 100 mg/kg/d and, at the same time, also was administered 5-fluorouracil (5-FU, 20 mg/kg/d) *via* intraperitoneal injection, twice a week; and another 6 nude mice served as a normal control group. After the treatment, researchers observed nude mice every day, weighed every two days, and measured the parameters of tumor growth every three days. Volume was determined based on these data, and the tumor volume growth curve was drawn with time in every group. After an intervention of 24 days, nude mice were sacrificed. Researchers severed carefully the tumor and weighed up for calculating the inhibition rate and tumor weight/body weight ratio, compared with the model group.

### 2.12. Immunohistochemistry Analysis

Apoptosis cell death was determined by in situ TUNEL analysis with the cell death detection kit for calculating the apoptosis rate, compared with the model group. Researchers randomly selected five nonoverlapping high-magnification horizons (×400) to count apoptotic and total cell numbers.

### 2.13. Statistical Analysis

GraphPad Prism 5.0 or SPSS 18.0 was employed for all statistical analyses.

Each experiment was carried out in triplicate. Results were expressed in the manner of mean ± SEM. One-way analysis of variance (ANOVA) was applied in pairwise comparisons between two groups. A difference of *P* < 0.05 was deemed statistically significant.

## 3. Results

### 3.1. *In Vitro* Anticancer Activity of WSTF

**Figure 2:**
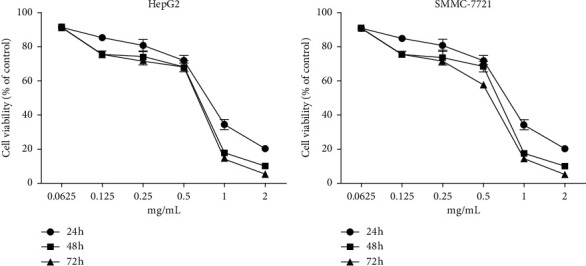
WSTF inhibits cancer cell proliferation in HepG2 and SMMC-7721 cells. Inhibitory effects of WSTF on the growth of cancer cells depending on the dose. MTT assay was carried out in cells exposed to WSTF for 24, 48, and 72 h. The results are presented in the manner of mean ± SEM from 3 independent tests. ^*∗*^*P* < 0.05, compared with vehicle-exposed controls.

The MTT assay was performed to evaluate the cytotoxic effects of WSTF on HepG2 and SMMC-7721 cells *in vitro*. It was illustrated from Figure 2 that, after WSTF treatment at the indicated doses for 24, 48, and 72 h, the cell numbers reduced depending on the WSTF dose, conforming to a prior work [24]. Based on these results of cytotoxicity (IC_50_) in Table 1, we further investigated apoptosis pathways.

### 3.2. WSTF Induce *G*0/*G*1 Phase Arrest and Increase ROS Levels

**Figure 3:**
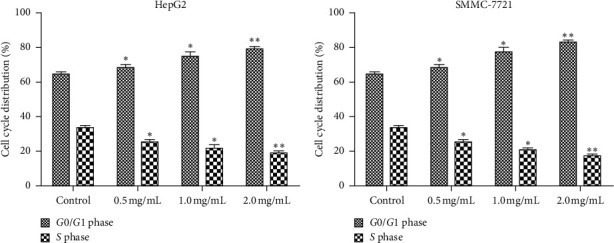
WSTF induces G0/*G*1 phase arrest. All results were presented in the manner of mean ± SEM from 3 independent tests. ^*∗*^*P* < 0.05 and  ^*∗∗*^*P* < 0.01 relative to controls.

**Figure 4:**
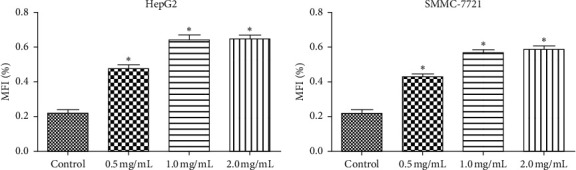
Effect of WSTF on ROS accumulation in SMMC-7721 and HepG2 cell lines. All results are presented in the manner of mean ± SEM from 3 independent tests. ^*∗*^*P* < 0.05 and  ^*∗∗*^*P* < 0.01 compared with controls.

We examined how WSTF affected the distribution of cell cycle of HepG2 and SMMC-7721 cells to analyze the mechanism of WFST inhibiting the growth of cancer cells. As shown in Figure 3, WSTF treatment led to the decreased proportion of cells at *S* phase and the increased proportion at *G*0/*G*1 phase, depending on the WSTF dose. These results indicated that WSTF may interfere with cancer cell growth through inducing *G*0/*G*1 phase arrest. As shown in Figure 4, WSTF treatment led to increased values of intracellular ROS (94.55%, 158.50%, and 166.20% in HepG2 cells and 116.95%, 192.41%, and 194.68% in SMMC-7721 cells, respectively, in comparison with control). Such results revealed that WSTF increased ROS accumulation.

### 3.3. NAC Reverses ROS Contents and Inhibits the WSTF-Induced Activation of Mitochondria-Dependent Apoptosis

**Figure 5:**
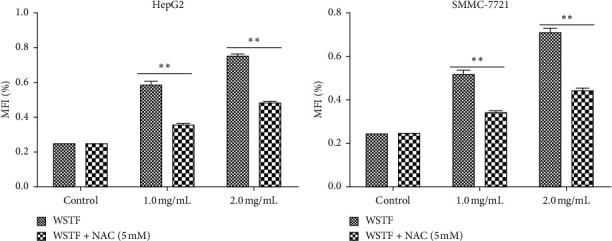
NAC inhibits the effect of WSTF on ROS accumulation in HepG2 and SMMC-7721 cell lines. Following 1 h of NAC treatment, cells were exposed to WSTF for 48 h. All results are expressed in the manner of mean ± SEM from 3 independent tests. ^*∗*^*P* < 0.05 and ^*∗∗*^*P* < 0.01 compared with controls.

**Figure 6:**
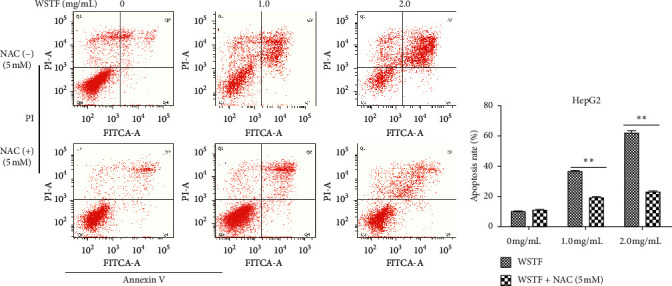
NAC inhibits apoptosis of HepG2 cells induced by WSTF. All results are presented in the manner of mean ± SEM from 3 independent tests. ^*∗*^*P* < 0.05 and ^*∗∗*^*P* < 0.01 relative to controls.

**Figure 7:**
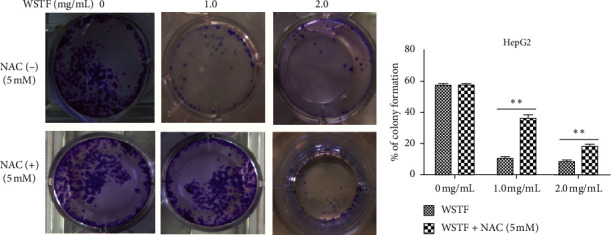
NAC inhibits the effect of WSTF on colony formation of HepG2 cells. After 1% crystal violet staining, each plate was photographed after 12 days. Then, the colony percentage was determined.

**Figure 8:**
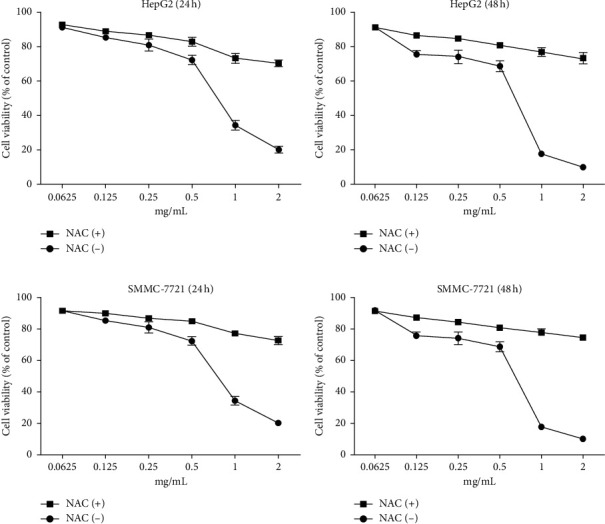
NAC weakens the inhibition of WSTF on cancer cell growth in HepG2 and SMMC-7721 cells. After 1 h NAC treatment, cells were exposed to WSTF treatment for 24 and 48 h. All results are expressed in the manner of mean ± SEM from 3 independent tests. ^*∗*^*P* < 0.05 and ^*∗∗*^*P* < 0.01 compared with controls.

The aim of our study is to investigate whether the increase in ROS induced by WSTF resulted in HCC cell apoptosis. After 1 h of NAC treatment (5 mM), cells were subjected to 48 h of WSTF exposure. It was observed from Figure 5 that preliminary NAC exposure resulted in an obvious decrease in ROS levels. This experiment showed that NAC inhibited apoptosis induced by WSTF (Figure 6). Another experiment showed that treatment with NAC clearly inhibited the effect of WSTF on colony formation (Figure 7). In addition, NAC weakened the inhibition of HCC cells by WSTF (Figure 8).

**Figure 9:**
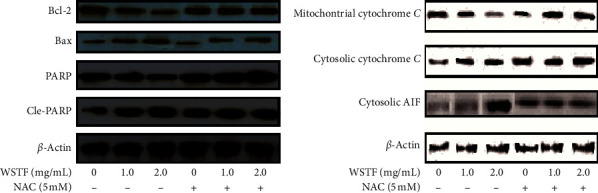
NAC inhibits the effect of WSTF on pro- and antiapoptotic protein levels. After 1 h of NAC pretreatment, HepG2 cells were exposed to WSTF for 48 h.

We applied western blot analysis to examine the related mechanisms by which WSTF initiated cell apoptosis. Results showed that WSTF induced decreased antiapoptotic protein (Bcl-2) levels but increased proapoptotic protein (Bax and PARP cleavage) levels (Figure 9(a)). Experiments showed WSTF weakened the relative cytochrome c band density within the mitochondria fraction, but the opposite tendency was seen within the cytosolic fraction. Besides, the results showed that WSTF increased the activity of cytosolic AIF (Figure 9(b)). These studies showed that WSTF-induced apoptosis was dependent on mitochondria.

However, NAC eliminated such alterations. We found out that NAC treatment changed those alterations observed in Bcl-2, Bax, PARP cleavage, and cytochrome c in the mitochondria fraction induced by WSTF (Figure 9(a) and 9(b)). Therefore, the findings showed that WSTF-induced mitochondria-dependent apoptosis was closely related to ROS accumulation.

### 3.4. WSTF Promote Anticancer Activity of 5-FU *In Vitro* Mechanism

**Figure 10:**
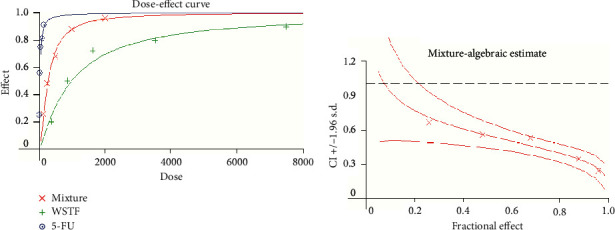
WSTF promote 5-FU anticancer activity *in vitro*. (a) 3000 cells/well were treated with WSTF plus 5-FU for MTT assay. The inhibition rates of WSTF and 5-FU on HepG2 cells were calculated. (b) CI values were calculated by CalcuSyn2.0.

**Figure 11:**
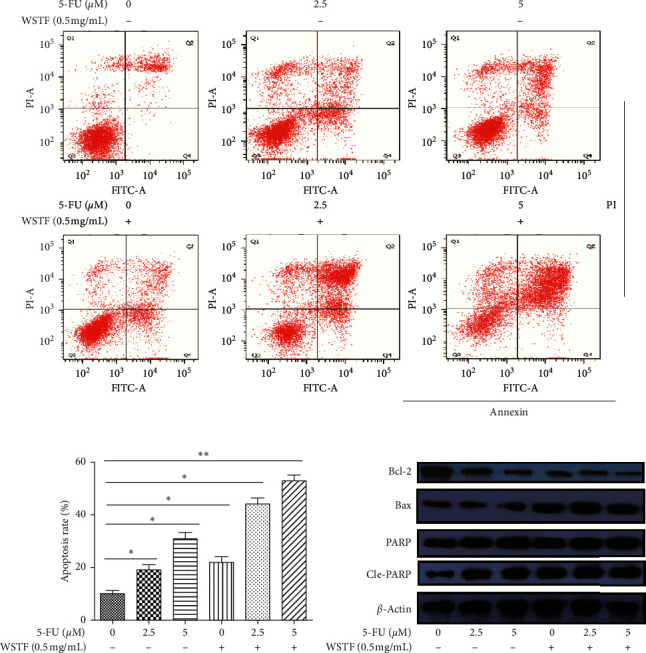
Combination of WSTF and 5-FU induces apoptosis. (a) HepG2 cells were subjected to Annexin V/PI staining for evaluating the WSTF and/or 5-FU-induced apoptosis. (b) Cell apoptosis percentages were expressed in the manner of mean ± SEM from 3 independent tests. (c) The expression of apoptosis-related proteins through Western blotting. ^*∗*^*P* < 0.05 and ^*∗∗*^*P* < 0.01.

To determine how WSTF plus 5-FU affected HCC *in vitro,* we evaluated the drug combination effects. As shown in Figure 10(a), there was a synergy in inhibiting the growth of HCC cells after the combination of WSTF and 5-FU. CI < 1 (Figure 10(b)) and DRI > 1 (Table 2) demonstrated, respectively, the synergistic effect of WSTF combined with 5-FU. That was to say, each drug dose was substantially reduced in the combination treatment relative to that in the single treatment (Table 2). As shown in Figure 11, WSTF combined with 5-FU markedly increased the apoptosis in treating HCC compared with 5-FU or WSTF single treatment that resulted in slightly increased apoptosis. Mechanism research by Western blot analysis also found an obvious increase in the expression of Bax and cleaved PARP after the combination of WSTF and 5-FU; on the contrary, the expression of Bcl-2 was reduced gradually (Figure 11(c)). The results suggest that WSTF promoted the anticancer activity of 5-FU *in vitro* by acting as a synergistic effect.

### 3.5. Combination Effects of WSTF plus 5-FU on HCC Cell Apoptosis and Colony Formation Capacity and Mechanism

**Figure 12:**
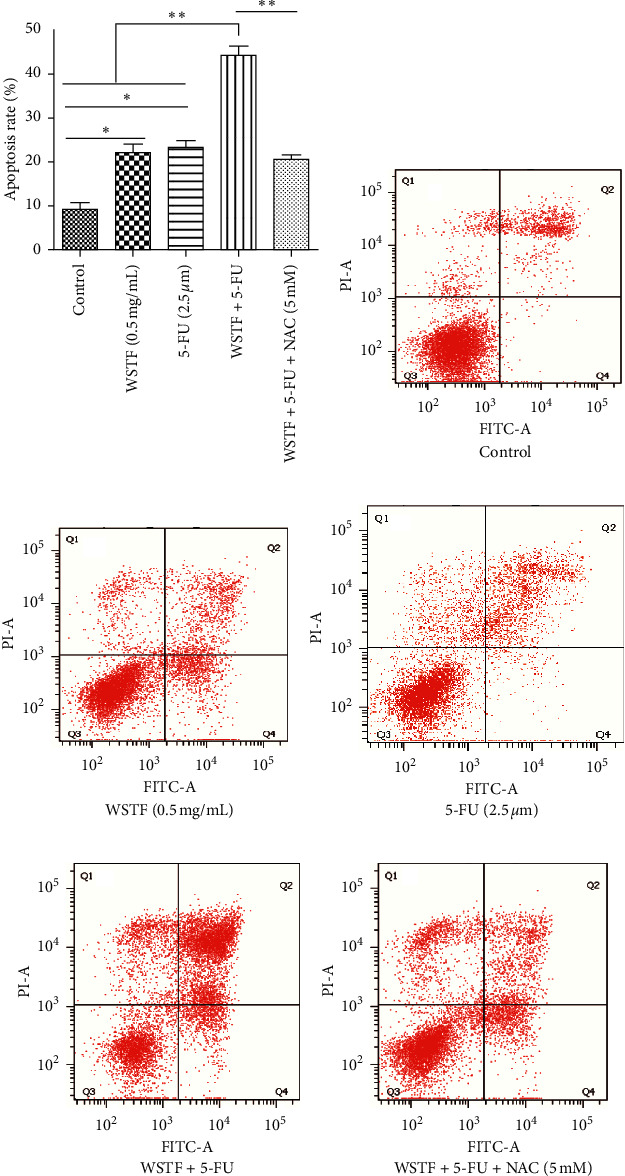
NAC inhibits the impact of WSTF plus 5-FU on apoptosis. HepG2 cells were subjected to Annexin V/PI staining for evaluating apoptosis induced by WSTF plus 5-FU with or without NAC.

**Figure 13:**
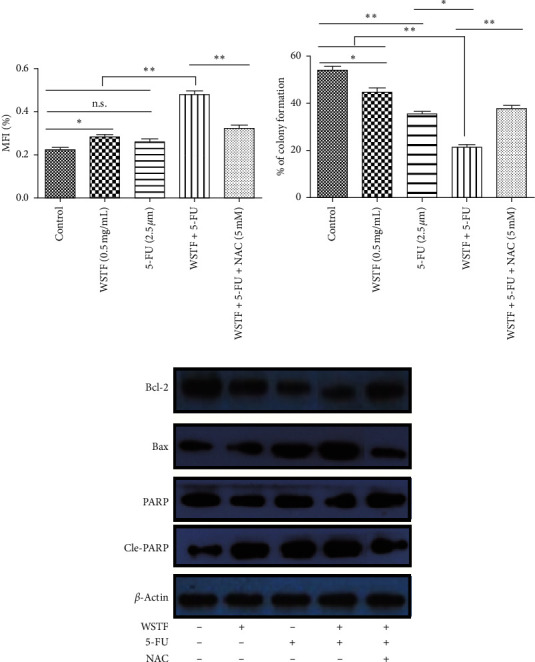
NAC inhibits the impact of WSTF plus 5-FU on ROS accumulation, colony formation, and apoptosis-related protein expression within HepG2 cells. (a) After 48 h of incubation with WSTF and 5-FU with or without NAC, the ROS level in HepG2 cells was determined by DCFH-DA, and the relative intensity of average fluorescence is presented in the manner of mean ± SEM from 3 independent tests. (b) After 48 h of incubation with WSTF and 5-FU with or without NAC, after 1% crystal violet staining, each plate was photographed after 10 days. Colony percentage was determined and presented in the manner of mean ± SEM from 3 independent tests. (c) After 48 h of incubation with WSTF and 5-FU with or without NAC, the expression of apoptosis-related proteins by Western blotting was completed. ^*∗*^*P* < 0.05 and ^*∗∗*^*P* < 0.01.

For investigating the related mechanisms of WSTF plus 5-FU in inducing apoptosis, this study explored colony formation, changes of ROS levels, and levels of proapoptotic and antiapoptotic proteins. It was illustrated from Figure 12 that the combination of WSTF and 5-FU upregulated apoptosis relative to each drug alone, whereas NAC pretreatment decreased apoptosis. Meanwhile, the combination of WSTF and 5-FU clearly decreased colony formation but significantly elevated ROS levels, but adding NAC weakened the synergy between WSTF and 5-FU (Figure 13(a) and 13(b)). As shown in Figure 13(c), mechanism research by Western blotting further revealed that WSTF plus 5-FU reduced Bcl-2 levels but elevated Bax and PARP cleavage levels, and the addition of NAC strongly recovered the expression levels of anti- or proapoptotic proteins resulting from WSTF and 5-FU. Inhibition of ROS accumulation reduced the synergistic effect of WSTF and 5-FU. These results suggest that WSTF combined with 5-FU had synergy on HCC cell apoptosis and colony formation through ROS accumulation *in vitro*.

### 3.6. WSTF Promote 5-FU Antitumor Activity *In Vivo*

**Figure 14:**
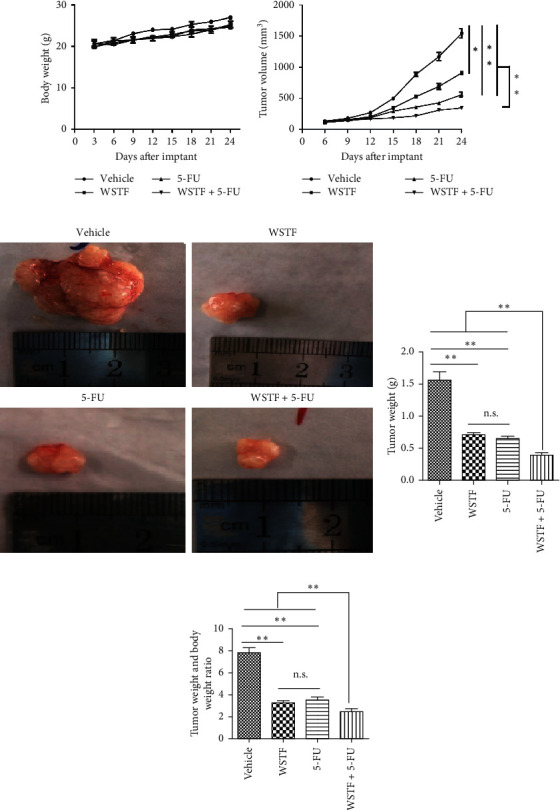
WSTF promote anticancer activity of 5-FU *in vivo*. (a) Body weight curves of mice in each group. Combination treatment with WSTF and 5-FU did not affect the nude mouse body weight. (b) Xenograft growth curve. Subcutaneous injection of HepG2 cells was given to mice through the oxter (xenograft volume was determined at intervals of 3 days) and drug administration started. (c) The specimen of xenografts among four groups. (d) Tumor weights of four groups. (e) Tumor/body weight ratio in the four groups. Results are expressed in the manner of mean ± SEM from 3 independent tests. ^*∗*^*P* < 0.05,  ^*∗∗*^*P* < 0.01, and n.s.: no significance.

**Figure 15:**
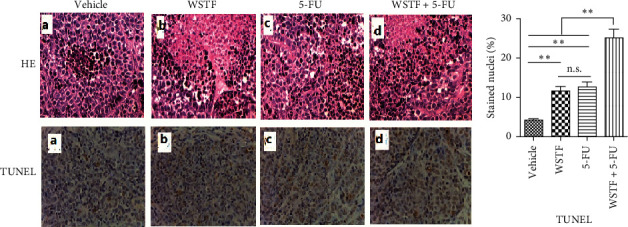
H&E staining. (a) Hematoxylin of HepG2 cell xenografts in nude mice (HE × 200) and TUNEL assay (×40) for xenografts. (b) TUNEL-stained nucleus percentage was used to evaluate apoptosis by TUNEL staining. Results are expressed in the manner of mean ± SEM from 3 independent tests. ^*∗*^*P* < 0.05,  ^*∗∗*^*P* < 0.01, and n.s.: no significance.

After 24 days of intervention, the body weight of mice and the tumor growth curve of tumor volume were drawn, respectively. As shown in Figure 14(a), the combination treatment with WSTF and 5-FU made no difference on nude mouse body weight. Studies showed that WSTF combined with 5-FU had significant effects on reducing the weight and volume of HCC tumors, as shown in Figure 14(b)–14(d). Experimental studies have found that nude mice in the 5-FU group are more thin, are lazy to move, eat less, and experience diarrhea because of the side effects of 5-FU. The most interesting is that the combination group has an advantage over the 5-FU group, that is, there is not only the reduction of the tumor weight but also the improvement of the tumor weight/body weight ratio clearly (Figure 14(e)). These findings implied WSTF combined with 5-FU in clinical practice could both inhibit tumor cell growth and overcome the side effects of 5-FU. Compared with vehicle, the results of tumor pathohistologic examination showed that WSTF, 5-FU, and combination groups showed obvious changes, respectively, such as tumor cell apoptosis and tissue heavy/medium necrosis (Figure 15(a)). What is interesting is that the combination group has a more significant increase in TUNEL-stained nuclei than WSTF and 5-FU groups (Figure 15(b)). These findings implied that WSTF combined with 5-FU significantly induced apoptosis, suppressing tumor cell growth while overcoming the side effects of 5-FU.

## 4. Discussion

Studies showed that WSTF could increase ROS accumulation and promote mitochondrial cytochrome c release into the cytoplasm, which may be related to the downregulation of Bcl-2 and survivin, as well as the upregulation of Bax and caspase-3 [25]. Yet most studies have focused on using WSTF as a potential drug alone. In this experimental study, we investigated whether WSTF have an inhibitory effect on cell growth and promote the anticancer effect of 5-FU both *in vivo* and *in vitro*. Based on these findings, WSTF suppressed cell proliferation through causing cell cycle arrest at the *G*0/*G*1 phase while inducing mitochondria-dependent apoptosis *via* ROS accumulation. Antioxidants can decrease ROS accumulation and relieve further oxidative stress in the cell [30]. In this experiment, NAC (N-acetylcysteine) was used as a tool drug (a potent antioxidant agent) to investigate the mitochondria-dependent apoptosis induced by WSTF. NAC can maintain the stability of TRX-ASK1 and P38 MAPK pathways [31]. The changes of ROS level and mitochondrial membrane potential are important factors in stimulating apoptosis [32]. Studies showed that the balance between increase and decrease of ROS played an important role in maintaining the normal biochemical state of the body [33] and the changes of ROS in cytoplasmic levels were essential for inhibiting tumor cell growth and development [34]. Our study showed that WSTF downregulated the Bcl-2 level but upregulated Bax and PARP cleavage levels. These findings indicated that WSTF stimulated HCC apoptosis through a mitochondria-dependent pathway. The presence of NAC can decrease the influence of WSTF on Bcl-2, PARP cleavage, and Bax levels. The reduction of NAC could correlate with inhibiting ROS accumulation. These results suggest that the WSTF-induced activation of apoptosis dependent on mitochondria was strongly correlated with the changes of ROS. In addition, WSTF also caused *G*0/*G*1 phase arrest. Accordingly, arrest at *G*0/*G*1 phase and cell apoptosis caused by WSTF are potential mechanisms to inhibit cancer cell proliferation.

In our study, WSTF could increase the accumulation of ROS, so we predicted that WSTF may enhance chemosensitivity to 5-FU through ROS-dependent pathways in the treatment of HCC. 5-FU is recognized as a more effective chemotherapy drug in the treatment of HCC [27, 35] and widely used for gastric cancer and colorectal cancer in clinical practice [26]. Compelling evidence showed that the adaptation of cells to ROS stress exerts a vital part in the maintenance of a cell phenotype of cancer and resistance to chemotherapeutic agents [36]. Studies have shown that accumulation of ROS enhanced chemosensitivity to 5-FU in MCF-7 cells [28]. We infer that WSTF may enhance chemosensitivity to 5-FU against HCC. For testifying the above hypothesis, this study calculated DRI and CI values of the combination treatment with WSTF and 5-FU to evaluate the feasibility of combination therapy. The study found that there was a synergy in inhibiting the growth of HCC cells after the combination of WSTF and 5-FU (CI < 1 and DRI > 1). WSTF combined with 5-FU surely played a significant role in promoting the accumulation of ROS, colony formation suppression, and apoptosis induction. Adding NAC reduced the synergy between WSTF and 5-FU by inhibition of colony formation, ROS accumulation, and apoptosis. The combination of WSTF and 5-FU decreased the Bcl-2 level but elevated PARP cleavage and Bax levels, while the addition of NAC strongly recovered the expression levels of anti- or proapoptotic proteins resulting from WSTF and 5-FU. These findings indicated that WSTF-increased ROS levels may partially or completely contribute to enhanced chemosensitivity to 5-FU. Finally, we used xenograft tumorigenicity assay to testify the synergistic effect on inhibition tumor growth *in vivo* after the combination of WSTF and 5-FU. Compared with single agents, combined treatment with WSTF and 5-FU significantly decreased tumor volumes that were consistent with the results *in vitro*. In our experiment, we also found that nude mice in the 5-FU group are more thin, are lazy to move, eat less, and experience diarrhea because of the side effects of 5-FU. The combination group has an advantage over 5-FU group, that is, there is not only a reduction in the tumor weight but also an improvement in the tumor weight/body weight ratio clearly. These findings implied WSTF combined with 5-FU in clinical practice could both inhibit tumor cell growth and overcome the side effects of 5-FU.

## 5. Conclusions

In a word, WSTF inhibit the growth of HCC and enhance chemosensitivity to 5-FU in hepatocellular carcinoma. WSTF-increased ROS levels may partially or completely contribute to enhanced toxicity. WSTF plus 5-FU for the treatment of HCC can play a synergistic effect in the clinical application.

## Figures and Tables

**Table 1:** Cytotoxicity of WSTF against normal human cells and tumor cells.

Cells	Time (h)	IC_50_ (mg/mL)
*HepG2*	24	0.52 ± 1.56
	48	0.42 ± 1.33
	72	0.36 ± 0.96
	24	0.73 ± 1.43

*SMMC-7721HCC*	48	0.59 ± 2.62
	0.40 ± 0.76	

Cells (5 × 10^4^ (ml)) were subjected to WSTF treatment at different doses under 37°C and 5% CO_2_ conditions for 24, 48, and 72 h, respectively. The cytotoxicity of WSTF was determined through MTT assay, which was presented in the manner of 50% inhibitory concentration (IC_50_). Data in each typical experiment were obtained from 3 independent tests.

**Table 2:** Effect of WSTF plus 5-FU on HepG2 cells.

Combination		Drug alone				DRI	
WSTF (mg/mL)	5-FU (*μ*M)	WSTF (mg/mL)	5-FU (*μ*M)	Fa	CI	WSTF (mg/mL)	5-FU (*μ*M)
0	0	0	0				
0.125	2.5	0.40 ± 0.14	7.20 ± 0.52	0.26 ± 0.02	0.66 ± 0.15	3.16 ± 0.62	2.88 ± 0.38
0.25	5	0.91 ± 0.34	17.58 ± 3.34	0.48 ± 0.05	0.56 ± 0.17	3.65 ± 0.77	3.52 ± 0.87
0.50	10	1.88 ± 0.63	38.00 ± 4.84	0.68 ± 0.08	0.53 ± 0.14	3.76 ± 0.95	3.80 ± 0.85
1.00	20	5.49 ± 1.03	119.42 ± 6.37	0.88 ± 0.09	0.35 ± 0.06	5.49 ± 1.34	5.97 ± 0.94
2.00	40	15.32 ± 2.32	357.37 ± 33.72	0.96 ± 0.07	0.24 ± 0.08	7.66 ± 1.26	8.93 ± 1.04

Fa is the fraction affected by the dose.

## Data Availability

The data used to support the findings of this study are included within the article.

## Abbreviations

WSTF: The water-soluble total flavonoids from *Isodon lophanthoides* var*. gerardianus* (Benth.) H. Hara
5-FU: 5-ﬂuorouracil
HCC: Hepatocellular carcinoma
DRI: Dose reduction index
CI: Combination index
TUNEL: TdT-mediated dUTP nick-end labeling
PARP: Poly ADP-ribose polymerase
DCFH-DA: 2,7-dichlorodihydrofluorescein diacetate.
